# Chemical–genetic attenuation of focal neocortical seizures

**DOI:** 10.1038/ncomms4847

**Published:** 2014-05-27

**Authors:** Dennis Kätzel, Elizabeth Nicholson, Stephanie Schorge, Matthew C. Walker, Dimitri M. Kullmann

**Affiliations:** 1 Department of Clinical and Experimental Epilepsy, UCL Institute of Neurology, Queen Square, London WC1N 3BG, UK

## Abstract

Focal epilepsy is commonly pharmacoresistant, and resective surgery is often contraindicated by proximity to eloquent cortex. Many patients have no effective treatment options. Gene therapy allows cell-type specific inhibition of neuronal excitability, but on-demand seizure suppression has only been achieved with optogenetics, which requires invasive light delivery. Here we test a combined chemical–genetic approach to achieve localized suppression of neuronal excitability in a seizure focus, using viral expression of the modified muscarinic receptor hM4D_i_. hM4D_i_ has no effect in the absence of its selective, normally inactive and orally bioavailable agonist clozapine-N-oxide (CNO). Systemic administration of CNO suppresses focal seizures evoked by two different chemoconvulsants, pilocarpine and picrotoxin. CNO also has a robust anti-seizure effect in a chronic model of focal neocortical epilepsy. Chemical–genetic seizure attenuation holds promise as a novel approach to treat intractable focal epilepsy while minimizing disruption of normal circuit function in untransduced brain regions or in the absence of the specific ligand.

## Abstract (web summary)

Focal epilepsy is difficult to treat with currently available drugs or surgical approaches. Kätzel *et al.* express mutant inhibitory receptors in the brains of rats with focal epilepsy and selectively activate these receptors by an exogenous compound, which results in region- and time-specific suppression of focal seizures

## Graphical abstract

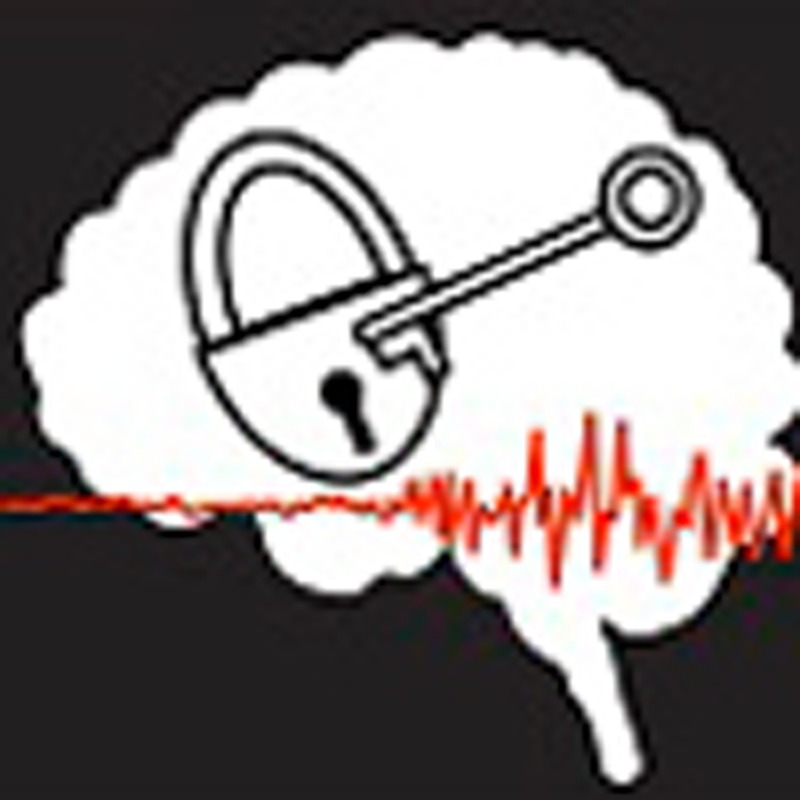

Epilepsy affects up to 1% of the population^1^ and is resistant to drug therapy in at least 20% of cases^2^. Epilepsy can be focal (arising from a specific brain area) or generalized (arising from both hemispheres). People with focal-onset epilepsy are especially prone to pharmacoresistance^3^. The epileptogenic zone in such cases is frequently restricted to a small region that can often be localized with imaging and electrophysiological techniques^4^. However, surgical removal of the seizure focus can successfully treat only about 5% of pharmacoresistant patients, and is often inappropriate in focal neocortical epilepsy because of proximity to eloquent cortex^5^,^6^.

Gene therapy targeted to the epileptogenic zone has been shown to be effective in rodent models of epilepsy including focal neocortical epilepsy^7^. However, viral delivery of transgenes that alter excitability permanently may impair essential function of circuits near the seizure focus. An attractive strategy would be to suppress circuit excitability ‘on demand’ upon detection of a seizure. Recently, progress in optogenetic seizure suppression in rodents has shown that this is, in principle, feasible^7^,^8^,^9^. One of the main limitations to clinical translation is the need to deliver light of the appropriate wavelength, intensity and duration to the region of transduced neurons. This necessitates the implantation of optical devices and suffers from the strong attenuation of light in brain tissue.

We report an alternative gene therapy approach to achieve targeted and temporally limited suppression of neuronal excitability that relies on systemic delivery of a small molecule inhibitor. Specificity is achieved by regional and cell-type specific expression of a designer receptor exclusively activated by a designer drug (DREADD). Neurons transduced with a DREADD are in principle unaffected in the absence of the selective ligand, and only affected when the ligand is present, thereby avoiding permanent alteration of their properties^10^. We chose the engineered inhibitory G_i_-coupled human muscarinic receptor hM4D_i_, which has been made sensitive to the orally bioavailable and normally inert metabolite of clozapine, clozapine-N-oxide (CNO)^11^,^12^. Importantly, hM4D_i_ is relatively insensitive to acetylcholine, the endogenous agonist of the parent receptor. hM4D_i_ activation leads to the opening of G-protein gated inwardly rectifying potassium channels, resulting in membrane hyperpolarization and neuronal inhibition^11^.

## Results

### Chemical–genetic silencing of pilocarpine-induced seizures

**Figure 1:**
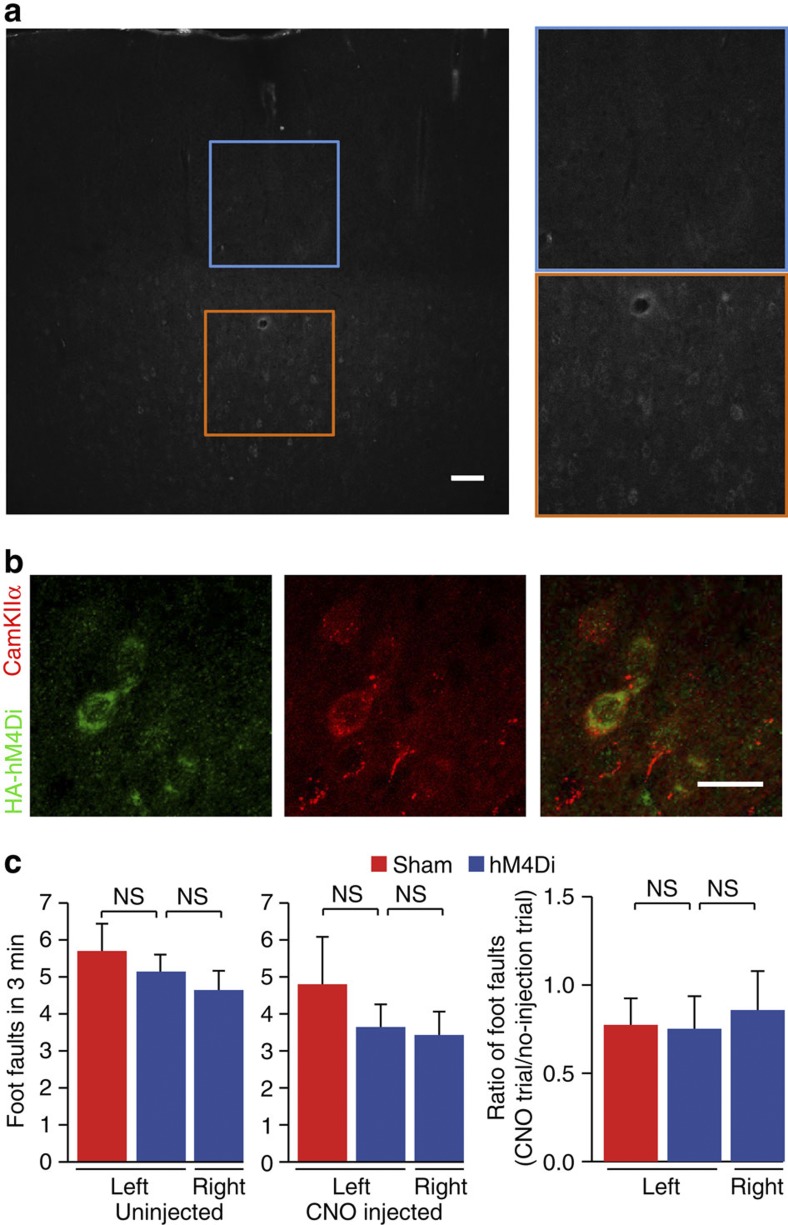
Expression and tolerability of HA-hM4D_i_-mCitrine. (**a**) Expression of HA-hM4D_i_-mCitrine in deeper layers of right primary motor cortex (M1), visualized with anti-HA antibody. Scale, 100 μm. (**b**) Colocalization of CamKIIα-HA-hM4D_i_-mCitrine, visualized with anti-HA (green) and anti-CamKIIα (red) antibodies. Scale, 20 μm. (**c**) Motor coordination with (right) and without (left) intraperitoneal CNO in rats receiving sham injection (red) or HA-hM4D_i_-mCitrine in right M1 (blue). Foot faults for the left forelimb (contralateral to HA-hM4D_i_-mCitrine) were compared with the right (ipsilateral) forelimb (paired *t-test*) and with foot faults of the left forelimb of sham-injected animals (unpaired *t-test*; mean±s.e.m., *n*=5 sham-operated and 7 hM4-injected rats). NS: *P*>0.05.

To test the ability of the DREADD to modify seizure activity, we injected an adeno-associated virus (AAV) encoding hM4D_i_ under the *CamkIIα* promoter (AAV5-CaMKIIα-HA-hM4D(G_i_)-IRES-mCitrine) into the forelimb area of primary motor cortex (M1) of rats weighing 263–325 g under isoflurane anaesthesia. At the same time we implanted both a Teflon cannula guide above the injection site to allow administration of chemoconvulsants and a subcutaneous transmitter (Open Source Instruments Inc.) with the active lead overlying M1 for wireless electroencephalography (EEG) recording. The transmitter samples the EEG at 512 Hz continuously for several weeks^13^. Expression of hM4D_i_ (Fig. 1a,b) had no detectable effect on behaviour or limb use (Fig. 1c), and was confirmed by fluorescence microscopy for all rats sacrificed after 4–20 weeks.

**Figure 2:**
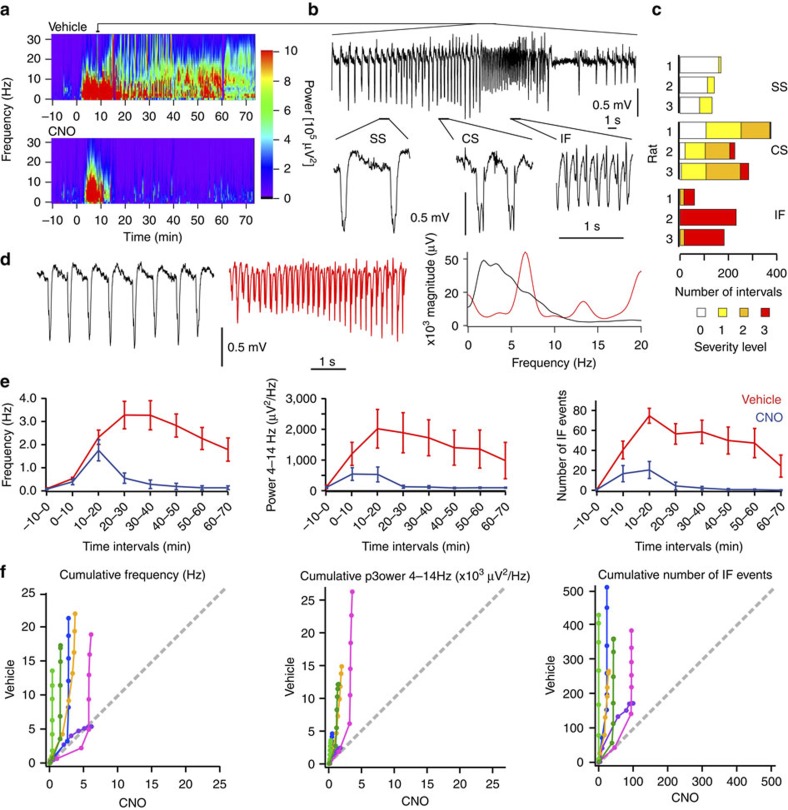
Chemical–genetic silencing of pilocarpine-induced seizures. (**a**) Morlet-wavelet EEG spectra from a rat administered intracortical pilocarpine (time 0), with either intraperitoneal vehicle (top) or CNO (bottom). (**b**) EEG segment from (a, top), showing simple spikes (SS), complex spikes (CS) and runs of intermediate frequency (IF) activity (expanded below). (**c**) Behavioural seizures correlated with EEG. SS activity was associated with no motor seizures (severity score 0) or twitching of limb, head or body (score 1), while IF was associated with repetitive head or body shaking (score 2) or rearing, retrograde locomotion and generalized convulsions (3). Six hundred consecutive 4-s-intervals per rat were assessed in three rats (numbered 1–3). (**d**) Detection of Intermediate Frequency (IF)-activity by Fourier transformation: Fourier transform (right) of EEG segments in one rat showing either SS (black) or IF (red) activity (two 5-s periods shown to the left) induced by pilocarpine. The IF Fourier transform shows a peak around 7 Hz (and a harmonic at 14 Hz). (**e**) Temporal evolution of spike frequency (left), 4–14 Hz power (middle), and number of IF runs (right), in vehicle (red) and CNO (blue) trials (consecutive 10-min-intervals before (-10–0 min) and after pilocarpine and vehicle/CNO injection). *N*=6 rats (10 pairs of trials, averaged within rat where repeated, data are shown as mean±s.e.m.). (**f**) Same data as in **e**, but plotted as cumulative electrographic seizure metrics (frequency, power, number of IF events), comparing vehicle versus CNO for each animal (indicated by colour). Symbols indicate consecutive cumulative metrics at 10-min-intervals. The 45-degree line (grey) indicates equivalence of CNO and vehicle.

We first examined seizures acutely evoked by chemoconvulsant injection into layer 5 of the motor cortex 17–52 days after hM4D_i_ AAV injection. Pilocarpine (5 M, 200–900 nl) injected via the implanted cannula guide (1.6–2.0 mm from skull surface) elicited large-amplitude spike-wave deflections at a frequency between 0.5 and 2 Hz, starting within 5 min of injection and lasting between 45 and 90 min (Fig. 2a,b). Spike-wave complexes either had a single negative peak (‘simple’ spike-waves, SS) or featured one or more shoulders (polyspike-waves or ‘complex’ spike-waves, CS; Fig. 2b). They were interspersed with runs of intermediate frequency (IF) discharges (5–12 Hz) lasting 0.2–12 s, which typically had a smaller amplitude (Fig. 2b). IF discharges correlated with the occurrence of more severe motor seizures. Motor seizures ranged from brief twitches of the contralateral limb, the head or the body (score 1), repetitive head, limb or body shaking (score 2), to rearing, retrograde locomotion and generalized convulsions lasting several seconds (score 3) (Fig. 2c). We therefore used the EEG power in an overlapping frequency band (4–14 Hz) as a surrogate marker to assess the anti-seizure effect of hM4D_i_ activation.

We randomly interleaved experiments on alternate days on which either CNO (1 mg kg^−1^ in dimethyl sulfoxide (DMSO)/saline vehicle)^12^, or vehicle alone, was administered by intraperitoneal injection immediately after focal neocortical pilocarpine infusion. In CNO trials, both electrographic and motor convulsions were substantially reduced (*n*=6 rats). Repeated measures analysis of variance (ANOVA) for the first six 10 min intervals post vehicle/CNO injection revealed a significant decrease in the mean frequency of negative deflections in the EEG, the mean 4–14 Hz power, and the number of IF runs that correlate with severe motor seizures (Fig. 2c,d; *P*<0.05). The seizure activity was on average larger in the vehicle trial than in the matched CNO trial for each rat and every 10 min interval (Fig. 2e,f).

### Chemical–genetic silencing of picrotoxin-induced acute seizures

**Figure 3:**
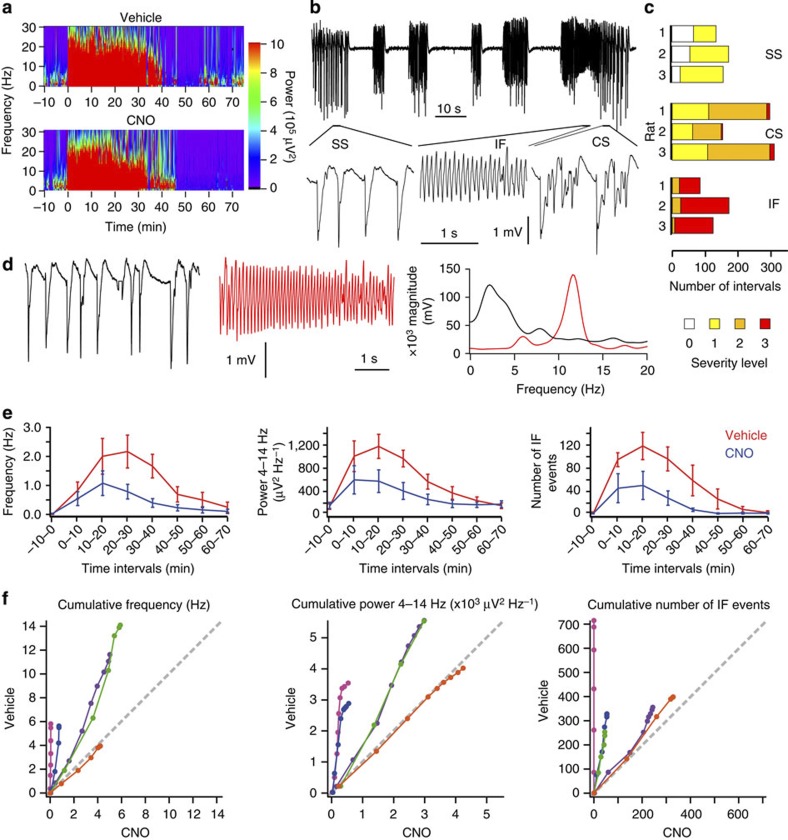
Chemical–genetic silencing of picrotoxin-induced seizures. (**a**) Morlet-wavelet power spectra of EEG in an animal injected with picrotoxin at time 0 (1 mm below pia, 10 mM, 300 nl), together with intraperitoneal vehicle (top) or 1 mg ml^−1^ CNO in vehicle (bottom) on consecutive days. (**b**) EEG activity. Bottom, expanded sections from times indicated (2-s-duration) showing SS, CS and IF activity. (**c**) Motor seizures were more severe in association with IF activity than with SS activity, and intermediate with CS activity (severity scale as in Fig. 2c). Six hundred consecutive 4-s-intervals per rat were assessed in three rats (numbered 1–3). (**d**) Fourier transform (right) of 5-s-traces displayed (left, middle) containing SS (black) and IF (4–14 Hz, peak around 11.5 Hz; red) activity induced by picrotoxin. (**e**) Temporal evolution of spike frequency (left), 4–14 Hz power (middle), and number of IF runs (right), in vehicle (red) and CNO (blue) trials (consecutive 10-min-intervals before (−10–0 min) and after picrotoxin and vehicle/CNO injection). *N*=5 rats (12 pairs of trials, averaged within rat where repeated, mean±s.e.m.). (**f**) Same data as in **e**, but plotted as cumulative electrographic seizure metrics for each animal as in Fig. 2e.

CNO thus profoundly suppressed pilocarpine-triggered seizures. However, the interpretation of this anti-seizure effect is potentially confounded by an overlap of downstream signalling cascades of pilocarpine acting on muscarinic receptors and CNO acting on hM4D_i_^14^. We therefore tested a second chemoconvulsant, the GABA_A_ receptor blocker, picrotoxin. Picrotoxin injection into the primary motor cortex (10 mM, 100–600 nl) also elicited electrographic and motor seizures. These were similar in overall duration, composition of spike-wave and IF complexes, and behavioural correlates (Fig. 3a–d) to seizures caused by pilocarpine. Among minor differences, electrographic bursting switched on and off more abruptly and individual polyspike-wave complexes lasted longer (Fig. 3b). When CNO was administered by intraperitoneal injection immediately after focal picrotoxin the electrographic discharges were again attenuated (Fig. 3e; *n*=5 rats; repeated measures ANOVA, *P*<0.05). This was especially marked for IF activity (Fig. 3e,f), which correlated with more severe motor seizures (Fig. 3c,d).

**Figure 4:**
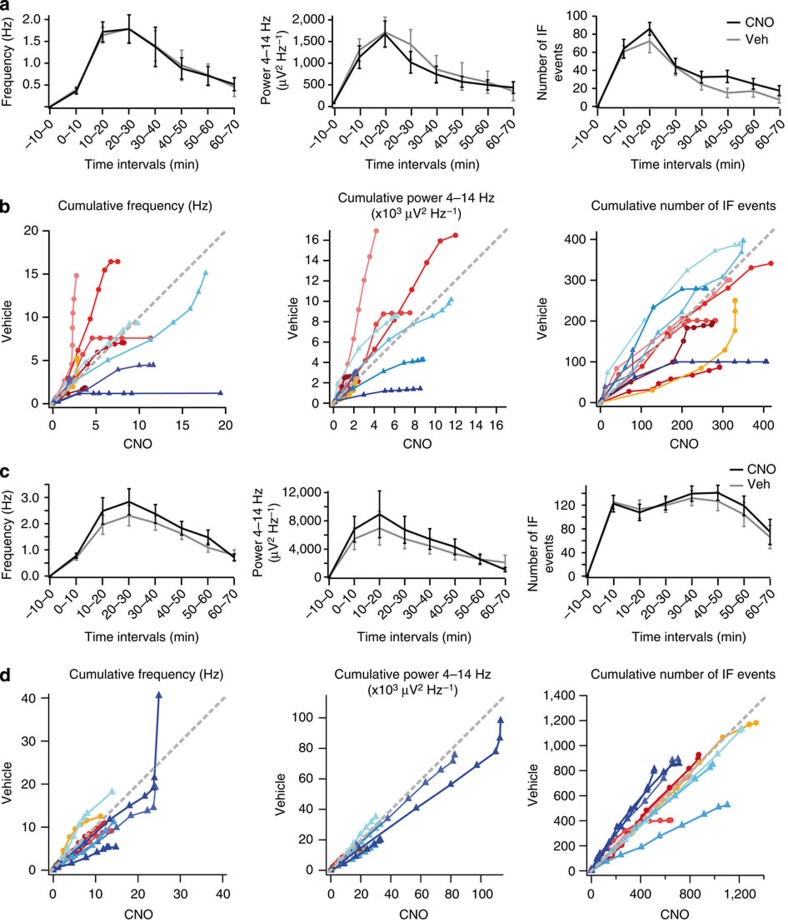
Effect of CNO on chemically induced seizures in control rats. Two distinct control groups per condition were tested: rats injected with an AAV5-CamKIIα-ArchT-GFP virus, and rats which were not injected with a virus (but otherwise underwent the same surgery and implantations as the other groups). For panels (**a**) and (**c**) data from both groups were pooled, while in panels (**b**) and (**d**) data from all rats and groups are shown individually. (**a**) Temporal evolution of spike frequency (left), 4–14 Hz power (middle), and number of IF runs (right), in vehicle (grey) and CNO (black) trials (consecutive 10-minute intervals before (—10–0 min) and after pilocarpine and vehicle/CNO injection. *N*=6 ArchT-virus transfected and five untransfected rats (two pairs of trials per animal, which were averaged within each rat; mean±s.e.m.). (**b**) Same data as in **a**, but plotted as cumulative seizure metrics for each individual animal (indicated by colour; blue hues with triangle symbols indicate untransfected animals, red hues with circles indicate ArchT-transfected animals; display as in Fig. 2f). (**c**) Temporal evolution of spike frequency (left), 4–14 Hz power (middle), and number of IF runs (right), in vehicle (grey) and CNO (black) trials (consecutive 10-min-intervals before and after picrotoxin and vehicle/CNO injection. *N*=5 ArchT-transfected and six untransfected rats (two pairs of trials averaged within each rat; one animal in the ArchT-group contributed only one pair of trials; mean±s.e.m.). (**d**) Same data as in **c**, but plotted as cumulative electrographic seizure metrics colour-coded as in **b**.

We asked whether off-target effects of CNO independent of hM4D_i_ could account for its anti-seizure effect. We conducted control experiments in both, rats injected with an analogous virus expressing the optogenetic actuator ArchT instead of hM4D_i_ as well as rats, which had no virus injected. Animals underwent the same experimental protocol as described above, using local intracortical injection of either pilocarpine (six virus-injected and five uninjected rats; Fig. 4a,b) or picrotoxin (five virus-injected and six uninjected rats; Fig. 4c,d). We observed no significant reduction of seizure activity in CNO compared with vehicle trials in any of the three measures of seizure severity in any of the control groups (repeated measures ANOVA, *P*>0.05).

**Figure 5:**
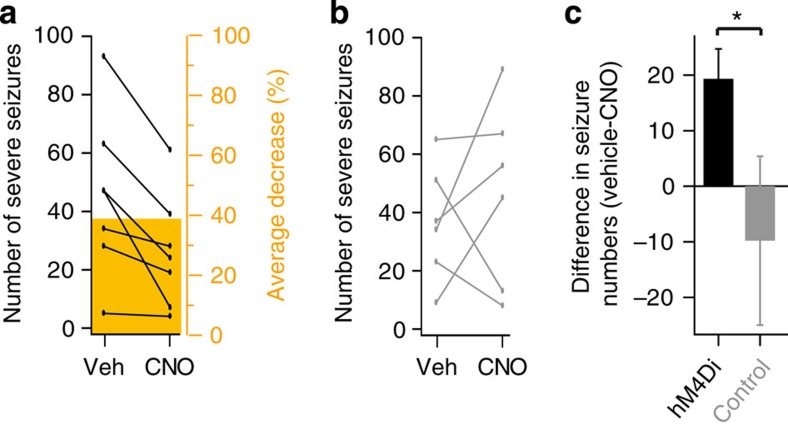
Chemical–genetic silencing of picrotoxin-induced motor seizures. (**a**,**b**) Pair-wise comparison of the number of episodes with severe motor seizures (class 3, as rated for Fig. 3c, see Methods) between vehicle and CNO trials in hM4D_i_-transfected (**a**, *n*=7) and untransfected (**b**, *n*=6) rats. Two pairs of trials were conducted per animal and the counts averaged within each animal. Orange bar indicates the decrease (%, right axis), where significant (paired *t-test*; *P*<0.05). (**c**) The absolute difference in the number of severe motor seizures for hM4D_i_-transfected (black) and untransfected rats (grey; error bars indicate s.e.m.; asterisk indicates statistical significance, *P*=0.031, one-tailed unpaired *t-test*).

Finally, we asked if chemical–genetic inhibition of focal seizure activity would result in a significant reduction of behavioural seizures. We focused on the picrotoxin model, where the anti-seizure effect of CNO/hM4D_i_ was less pronounced, to establish a conservative benchmark. Rats (*n*=7) underwent a similar treatment as before, but the virus was left to express for 3 months before the first experiment was conducted to achieve high expression levels. Seizures of the most severe class (score 3, see above and Methods) were counted by an experimenter who was blind to the identity of the injected compound (vehicle versus CNO, 4 mg kg^−1^). The number of severe behavioural seizures was reduced in CNO trials relative to vehicle trials in all subjects (*P*=0.012, paired *t*-test), with an average decrease of 39.5±8.7% (mean±s.e.m., Fig. 5a) or 19.3 seizure episodes (Fig. 5c). No effect was observed in control animals, which were not injected with virus (*n*=6; *P*=0.510, paired *t*-Test, Fig. 5b).

### Chemical–genetic silencing of focal neocortical epilepsy

**Figure 6:**
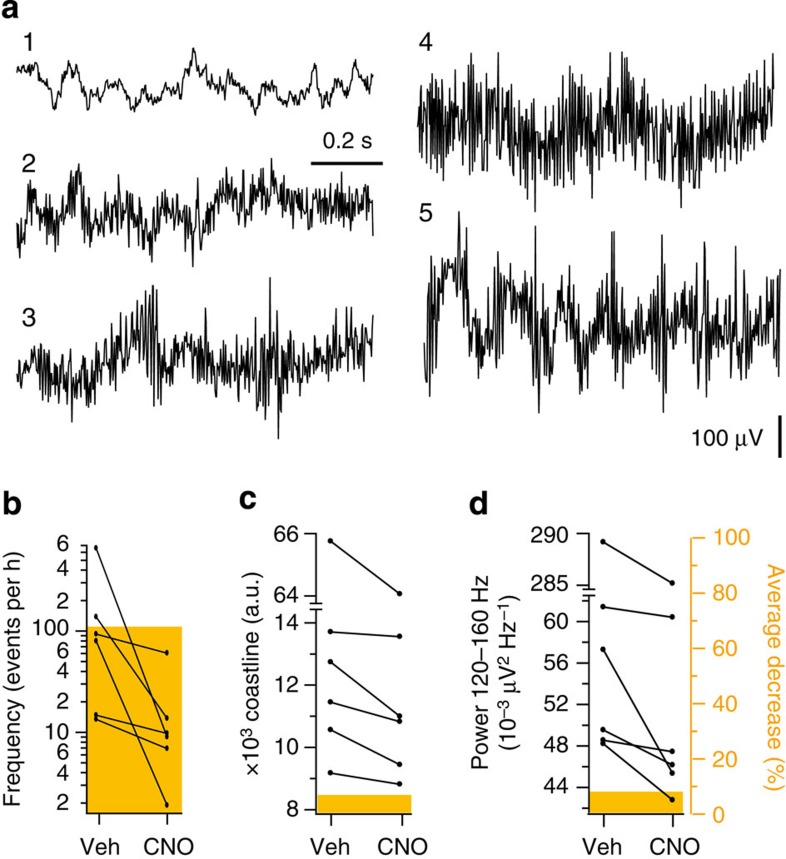
Chemical–genetic silencing of focal neocortical epilepsy. (**a**) Sample EEG traces from a tetanus toxin injected animal, showing background activity (1) and four types of epileptiform activity: ‘long events of low amplitude’ (2), ‘short events of high amplitude’ (3), ‘long event of high amplitude’ (4), and ‘high amplitude plus intermittent spikes’ (5) (see ref. 7). (**b**–**d**) Pair-wise comparison of the frequency of epileptiform events (**b**), coastline (**c**) and high-frequency power (**d**) between vehicle and CNO trials. *N*=6 rats; 15 pairs of trials, averaged within animal where repeated. Orange bars indicate the decrease (%, right axis), where significant (Wilcoxon test; *P*<0.05).

hM4D_i_ activation with CNO is thus effective in two chemoconvulsant models. Does it also suppress spontaneous seizures in established epilepsy? We turned to the tetanus toxin model of chronic epilepsy^15^,^16^, which responds poorly to antiepileptic drugs and resembles human epilepsia partialis continua^17^. This model is characterized by several EEG features, including increased high-frequency (120–160 Hz) power, increased coastline (cumulative difference between successive points on the EEG) and the occurrence of brief bursts of high-frequency EEG activity that can be detected by an automated event classifier^7^ (Fig. 6a).

Although the half-life of CNO in rats has not been measured systematically, it affects neurons transduced with hM4D_i_ or its excitatory analogue hM3D_q_ for at least 90 min^12^,^18^,^19^, and is fully cleared within 12 h of administration of its precursor clozapine^20^. We therefore assessed electrographic markers of epilepsy during a 3.5-h window starting with the first of two intraperitoneal injections of either CNO or vehicle, with the second injection at 2 h. The assessment was then repeated 24 h later, switching vehicle and CNO, to allow for washout of the agonist, and to control for diurnal variability in seizure frequency (Fig. 6b–d, six rats). All three measures of epilepsy (frequency of epileptiform bursts, coastline and high-frequency power) were significantly attenuated by CNO when compared with vehicle (*P*=0.028, Wilcoxon test; *n*=6).

### hM4D_i_ inhibits synaptic transmission

**Figure 7:**
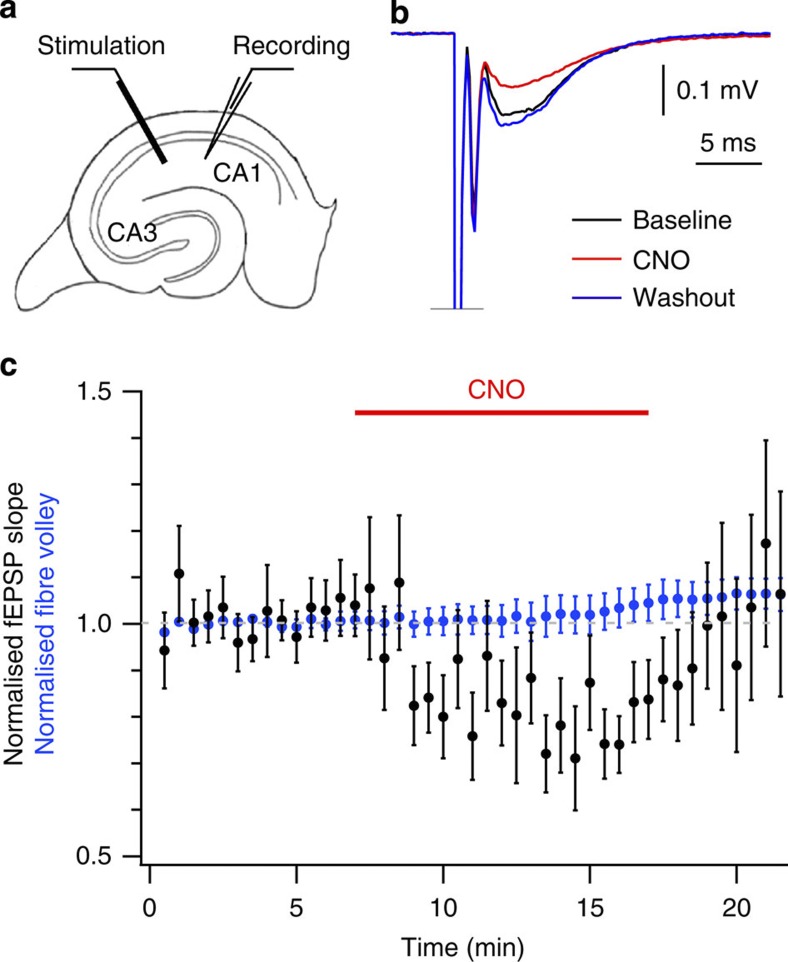
Chemical–genetic inhibition of synaptic transmission. (**a**) Experimental configuration; a stimulation electrode was placed in stratum radiatum to activate Schaffer collateral fibres expressing HA-hM4D_i_-mCitrine. The resulting fEPSP, evoked every 30 s, was recorded further medial in CA1 with an extracellular electrode. (**b**) Sample traces showing a stimulation artifact, fibre volley and subsequent fEPSP under baseline condition (black), during CNO wash-in (10 μM; red) and after washout (blue). (**c**) Average normalized slope of evoked fEPSPs (black) and average normalized amplitude of evoked fibre volleys over time before (6 min), during (10 min) and after (6 min) CNO (10 μM) across slices (*n*=9). Slices were only included if mCitrine fluorescence was visible in CA3 and CA1. Error bars show s.e.m.

While the optogenetic approach of on-demand silencing of epilepsy relies on direct hyperpolarization via outward currents, the more indirect, chemogenetic inhibition via hM4D_i_ may recruit at least two strategies of neuronal inhibition: first, activation leads to the opening of G-protein gated inwardly rectifying potassium channels, resulting in membrane hyperpolarization^11^. Second, synaptic transmission from excitatory cortical synapses might be directly inhibited, as has been shown for the native muscarinic receptor M4, which is expressed presynaptically^21^,^22^. While the former hyperpolarizing effect has been repeatedly documented for the modified receptor hM4D_i_, it is unclear if its potential for synaptic silencing may also contribute to the inhibitory effect we observe. We injected the hM4D_i_-expressing AAV into the CA3 subfield of the hippocampus in three rats under isoflurane anaesthesia at 4 weeks of age, and prepared acute hippocampal slices 10–11 weeks later. A field excitatory postsynaptic potential (fEPSP) was evoked in stratum radiatum of CA1 by extracellular stimulation. Bath perfusion of CNO (10 μM) reversibly decreased the initial slope of the fEPSP without affecting the fibre volley, consistent with a decrease in glutamate release (*n*=9 slices; Fig. 7). Although the depression was modest, this experiment underestimates the effect of hM4D_i_ activation on individual synapses because not all stimulated axons were supplied by transduced neurons. The inhibition of synaptic transmission likely contributes to the overall antiepileptic effect of hM4D_i_.

## Discussion

This study shows that chemical–genetics offers the prospect of attenuating seizures on demand. We demonstrate the effectiveness of this treatment in two focal seizure models and one model of focal neocortical epilepsy. Silencing was particularly pronounced when seizures were elicited by focal pilocarpine injection, but slightly less effective when picrotoxin was injected. This difference might reflect the loss of endogenous GABA_A_ receptor-mediated inhibition in the latter model, making it more difficult for the network to stabilize when excitability is reduced. Transduction with hM4D_i_ has no effect on neuronal excitability in the absence of its specific ligand CNO^10^,^11^,^12^, and so this approach avoids the theoretical risk of gene therapies designed around permanent overexpression of ion channels, neurotransmitter receptors or neuropeptides. Its temporal specificity does not match that of optogenetics^7^,^8^,^9^ because the duration of effect is dictated by the half-life of CNO, which has been estimated in humans at 7–8 h^23^. However, chemical–genetics avoids the need for invasive and biocompatible devices to deliver light to the transduced brain area close to the seizure focus. Moreover, a relatively large area may be targeted, which is not limited by absorption of light. Instead, CNO can be administered systemically.

We observed a significant reduction in seizure severity within 10 min of CNO administration (Figs 2e,f and 3e,f), well below the 30-min timepoint that usually defines status epilepticus. Many patients with drug-resistant epilepsy have seizures that are preceded by premonitory auras, or cluster at predictable times (for example, catamenial epilepsy), and hence might benefit from such chemical–genetic silencing. CNO’s bioavailability implies that it could be administered buccally or intranasally. For even faster on-demand administration, subcutaneous pumps, as used to deliver insulin^24^, could, in principle be used in people with epilepsy. Recent evidence that seizures can be predicted by automated EEG analysis^25^ offers the prospect of a closed-loop device.

A further potential application of chemical–genetics to epilepsy is to test the hypothesis that continued alteration of neuronal excitability for a fixed period might ‘reset’ epileptogenic circuits in some circumstances, bringing about a persistent reduction in seizures that outlasts the administration of the ligand. The reversibility, together with the regional and cell-type specificity of chemical–genetics, distinguishes this approach from available small molecules or gene therapies based on expression of ion channels or other signalling molecules.

In conclusion, we have shown that chemical–genetics is a promising approach to achieve region- and cell-type specific attenuation of neuronal excitability to suppress seizures. The pathway to translation is likely to be more direct than for optogenetics.

## Methods

AAV of serotype 5 containing a CamkIIα-HA-hM4D(G_i_)-IRES-mCitrine cassette provided by Dr Bryan Roth (University of North Carolina, UNC) was obtained from UNC Vector Core at a concentration of 8 × 10^12^ infectious units (IU) per ml. For control experiments (Fig. 4) a similar virus was injected expressing the optogenetic silencer ArchT instead of the chemical–genetic silencer hM4D_i_ (AAV5-CamkIIα-ArchT-GFP).

### Stereotactic surgery

Animal experiments were conducted in accordance with the Animals (Scientific Procedures) Act 1986, and approved by the local ethics committee. Male Sprague–Dawley rats (6–12 weeks old, 263–325 g) were anaesthetized using isoflurane and placed in a sterotaxic frame (Kopf, CA, USA). hM4D_i_-expressing AAV5-virus (1.5 μl) was injected at 100 nl min^−1^ into layer 5 of the forelimb area of right primary motor cortex (coordinates, 2.2–2.4 mm lateral and 1.0 mm anterior of bregma at a depth of 1.0 mm from pia; in some rats half of the volume each was deposited at 1.1 and at 0.7 mm from pia). An EEG transmitter (A3019D, Open Source Instruments^13^) was implanted subcutaneously with a subdural intracranial recording electrode positioned above the injection site. A reference electrode was implanted in the contralateral skull. For sequential injections of chemoconvulsants a Teflon cannula guide (C313GT/SP, PlasticsOne) was implanted above the injection site. For chronic epilepsy experiments, 12–16 ng of tetanus toxin (gift of Dr G. Schiavo) was injected together with hM4D_i_-expressing AAV5 virus in a final volume of 1.6–1.8 μl. Animals were housed separately in Faraday cages and EEG was recorded continuously for up to 8 weeks post surgery. Animal numbers per cohort were chosen to allow for detection of a therapeutic effect, while avoiding unnecessary procedures given their severity level.

### Assessment of motor coordination

Rats were either injected with AAV5-CamkIIα-HA-hM4D(Gi)-IRES-mCitrine unilaterally into the right-motor cortex (as described above) or underwent sham surgery (cut and suturing of skin). After 25–32 days, rats were placed on an elevated metal grid for 3 min. Two observers, blind to whether the rat had undergone sham surgery or hM4Di-injection, counted left and right forelimb foot faults. These were defined as the whole foot falling through the space between painted metal wires that made up the grid, spaced 4 cm apart. Four hours later, all rats were injected with CNO (1 mg kg^−1^) and the behavioural assessment repeated 25–33 min post-injection. Typically, the number of foot faults was lower on the second assessment as a result of training and habituation^7^. Two months later, the same rats were assessed in a similar manner, but with the reverse order of treatment: the first trial was conducted 24–37 min after injection of CNO, while the second trial was conducted ~\n5.5 h later without prior injection. Data from the two CNO and the two non-injection trials were each averaged for analysis.

### Brain slice experiments

AAV5-CamkIIα-HA-hM4D(G_i_)-IRES-mCitrine virus (1 μl) was injected into the CA3 subfield of the dorsal hippocampus of 4-week-old male Sprague–Dawley rats at 3.6 mm lateral (right), 2.8 mm posterior and 2.9 mm ventral from bregma at 100 nl min^−1^ (ref. 26). Ten to eleven weeks later, the animals were transcardially perfused with a room temperature solution containing (in mM): N-Methyl-D-glucamine-Cl, 92; KCl, 2.5; NaH_2_PO_4_, 1.25; Thiourea, 2; Ascorbic acid, 5; Na-Pyruvate, 3; MgCl_2_, 10; D-Glucose, 25; NaHCO_3_, 30; CaCl_2_, 0.5; Sucrose, 1 and horizontal hippocampal slices were prepared. The extracellular perfusion solution contained (in mM): NaCl, 119; KCl, 2.5; CaCl_2_, 2.5; MgSO_4_, 1.3; NaH_2_PO_4_, 1.25; NaHCO_3_, 25; and Glucose, 10. A fEPSP was evoked every 30 s by extracellular stimulation (20–320 μA, 100 μs) in stratum radiatum of CA1, before, during and after bath perfusion of CNO (10 μM).

### Seizure models

Pilocarpine (5 M in sterile saline) or picrotoxin (10 mM in 10% DMSO/sterile saline) were injected through the previously implanted Teflon cannula guide 17–52 days after hM4D_i_ AAV injection. The volume injected was adjusted between 200 and 900 nl for pilocarpine and 100–600 nl for picrotoxin, guided by the severity of the resulting seizures in each animal, and were kept constant between matched CNO and vehicle trials. CNO (1 mg kg^−1^, diluted at 1 mg ml^−1^ or 0.5 mg ml^−1^ in 1% DMSO/saline vehicle) or vehicle alone were injected intraperitoneally immediately after convulsant infusion. Spike-waves developed within 5 min of chemoconvulsant injection. For assessment of the inhibitory effect of hM4D_i_ activation on picrotoxin-induced behavioural (motor) seizures, experiments were conducted no earlier than 3 months after viral transfection and at a dose of 4 mg kg^−1^ CNO (in 4% DMSO/saline vehicle). The experimenter, who graded (see below) and counted the seizures over 60 min starting 5 min after CNO/vehicle injection, was blind to the identity of the injected compound.

### Epilepsy model

Tetanus toxin (12–16 ng) injected in a suspension with the hM4D_i_-expressing virus (see above), evoked high-frequency (70–160 Hz) events typically lasting less than 1 s, starting within 4 days. Such events occurred for up to 8 weeks after injection, but their frequency varied from day to day, and also depending on the time of day. Therefore, pairs of CNO/vehicle trials were matched according to time of day, and we set a criterion that at least two events per hour had to occur on an average over the 4 h before the first CNO or vehicle injections for the trial to be included in the dataset. The order of CNO and matched vehicle trials was randomized. Vehicle (1% DMSO/saline) or CNO (1 mg kg^−1^ in DMSO/saline) injected twice at 2-h intervals, and periods lasting 3.5 h after the first injection were analyzed.

### EEG analysis

EEG was recorded and processed using the Neuroarchiver tool (Open Source Instruments) and IgorPro (Wavemetrics Inc). The trace was centred around 0 V by subtraction of the average. Short (<100 ms), high-amplitude artifacts (‘glitches’) detected by threshold and periods with failed transmission were removed. The Igor script ‘UnipolarPeakAreas.ipf’ was used to detect individual negative deflections (spike-waves), and custom-written scripts in Igor extracted their frequency as well as the coastline and power of the trace. The coastline was determined as the sum of the absolute difference between successive points. EEG epochs were also exported into Labview (National Instruments) to compute Morlet-wavelet power spectra.

### Behavioural seizure analysis

To establish a correlation between different types of EEG-patterns and behaviour, we compared online scoring of seizures with EEG traces analyzed offline. Behaviour was also video-recorded for reference. EEG traces of 40 min length, starting 2–3 min after convulsant infusion, were divided into 4-s-intervals, and each interval was assigned a type of electrophysiological as well as behavioural activity. Electrophysiological activity was scored as simple or complex spike-waves, or as IF activity as described above (intervals without any spike-waves were not counted and never coincided with behavioural convulsions). Motor seizures were graded on a severity scale as follows: 0 (no obvious motor seizure), 1 (individual twitches of the contralateral limb or the head), 2 (repetitive shaking of forelimb, head or body), 3 (whole body shaking, arching and rearing sometimes accompanied by retrograde locomotion).

### Automated epileptiform event counting

For tetanus toxin-induced epileptic events, event sorting was based on six metrics extracted from each 1 s EEG epoch and compared with a library of EEG events, which had previously been classified as corresponding to seizures or artefacts^7^. The event-classifying routine stepped through consecutive epochs, and measured baseline power as the lowest power between 4 and 160 Hz in any 1 s epoch during the preceding 20 min. Epochs whose power exceeded 5 × baseline were defined as putative events. For each such event, the following six parameters were determined: Power (in the 4–160 Hz band), transient power (power in the 1–4 Hz band), high-frequency power (60–160 Hz), spikiness (voltage range/standard deviation), voltage asymmetry (balance of points exceeding two standard deviations on either side of the mean), and intermittency (low-frequency power of the rectified high-frequency signal). A sigmoidal function was applied to these six characteristics so as to obtain metrics bounded between zero and one. The event library was constructed by an operator who, with reference to synchronized video recordings, classified events as ‘no event’ (no obvious electrographic or behavioural event), ‘short-high-frequency bursts’ (<250 ms), ‘long-high-frequency bursts’ (>250 ms, event power >6 × baseline), ‘long-high-frequency bursts of low amplitude’ (>250 ms, event power 5–6 × baseline), ‘high-frequency spikes’, ‘eating-related’ or ‘grooming-related’. As the algorithm stepped through the subsequently identified events these were provisionally identified as belonging to one or the other category according to its Euclidean distance to previously classified neighbours. During the establishment of the library, the identity of each new event proposed by the algorithm was overruled by the observer if necessary until it reached a false allocation rate <1%. Once this criterion was satisfied, all EEG data were classified without further operator interference. A more complete description of the seizure detection algorithm with source code is available at: http://www.opensourceinstruments.com/Electronics/A3018/Seizure_Detection.html#Similarity%20of%20Events.

IF oscillations evoked by picrotoxin or pilocarpine were detected by using the fast-Fourier transform of 3-s EEG segments in the range 5–14 Hz (see also Fig. 2d). The 3-second window was moved along the trace in 1-s steps. IF events were defined as periods where the peak magnitude between 5–14 Hz exceeded a threshold of 20–45 mV, depending on the overall intensity of activity. Thresholds were kept constant within each matched pair of CNO/vehicle trials.

### Statistical analysis

Paired *t*-tests or Wilcoxon tests, or unpaired *t*-tests were performed as appropriate using SPSS 20 (IBM). For seizure models, experimental time was divided into 10-min periods including the 10 min before injection as well as seven consecutive 10-min periods after injection, and treatment effects were assessed with repeated measures ANOVA.

### Fluorescence and immunohistochemical analysis

Brains were removed and left in 4% paraformaldehyde/phosphate-buffered saline (PBS) for 3–7 days at 4 °C and then washed in PBS. Coronal slices (50 and 100 μm thickness) were cut on a vibrating slicer and examined for native mCitrine-expression right after slicing for each rat contributing to the dataset. Some of the 50 μm slices were processed further: they were permeabilized in PBS, 0.15% Triton X-100 for 20 min, blocked with 10% horse serum (Vector Labs) for 1 h on a shaker and incubated for 2 days in primary antibodies against CaMKIIα (rabbit, 1:500, Epitomics/Abcam) and haemagglutinin (mouse, 1:1,000, Covance). Following three further washes in PBS (10 min), the sections were incubated in secondary antibodies (1:500, Invitrogen, labelled with Alexa-488 and Alexa-546) overnight at 4 °C, washed in PBS again (four times, 10 min) and mounted in Vectashield (Vector Labs). Images were obtained with a confocal microscope at 25 × magnification of the objective and 3 × digital magnification.

## Author contributions

D.K. performed *in vivo* experiments. E.N. performed *in vitro* experiments. D.M.K., S.S. and E.N. assisted with behavioural experiments requiring blind assessors. D.K. and D.M.K. designed the study and wrote the manuscript. D.K., D.M.K., E.N. and M.C.W. analyzed data. S.S., M.C.W. and D.M.K. provided rodent telemetry facilities. All authors revised the manuscript.

## Additional information

**How to cite this article:** Kätzel, D. *et al.* Chemical–genetic attenuation of focal neocortical seizures. *Nat. Commun.* 5:3847 doi: 10.1038/ncomms4847 (2014).

## References

[b1] Ngugi A. K., Bottomley C., Kleinschmidt I., Sander J. W. & Newton C. R. Estimation of the burden of active and life-time epilepsy: a meta-analytic approach. Epilepsia 51, 883–890 (2010). 20067507 10.1111/j.1528-1167.2009.02481.x PMC3410521

[b2] Kwan P., Schachter S. C. & Brodie M. J. Drug-resistant epilepsy. N. Engl. J. Med. 365, 919–926 (2011). 21899452 10.1056/NEJMra1004418

[b3] Annegers J. F., Hauser W. A. & Elveback L. R. Remission of seizures and relapse in patients with epilepsy. Epilepsia 20, 729–737 (1979). 499118 10.1111/j.1528-1157.1979.tb04857.x

[b4] Rosenow F. & Lüders H. Presurgical evaluation of epilepsy. Brain J. Neurol. 124, 1683–1700 (2001). 10.1093/brain/124.9.1683 11522572

[b5] Schuele S. U. & Lüders H. O. Intractable epilepsy: management and therapeutic alternatives. Lancet Neurol. 7, 514–524 (2008). 18485315 10.1016/S1474-4422(08)70108-X

[b6] De Tisi J. *et al.* The long-term outcome of adult epilepsy surgery, patterns of seizure remission, and relapse: a cohort study. Lancet 378, 1388–1395 (2011). 22000136 10.1016/S0140-6736(11)60890-8

[b7] Wykes R. C. *et al.* Optogenetic and potassium channel gene therapy in a rodent model of focal neocortical epilepsy. Sci. Transl. Med. 4, 161ra152 (2012). 10.1126/scitranslmed.3004190 PMC3605784 23147003

[b8] Paz J. T. *et al.* Closed-loop optogenetic control of thalamus as a tool for interrupting seizures after cortical injury. Nat. Neurosci. 16, 64–70 (2013). 23143518 10.1038/nn.3269 PMC3700812

[b9] Krook-Magnuson E., Armstrong C., Oijala M. & Soltesz I. On-demand optogenetic control of spontaneous seizures in temporal lobe epilepsy. Nat. Commun. 4, 1376 (2013). 23340416 10.1038/ncomms2376 PMC3562457

[b10] Pei Y., Rogan S. C., Yan F. & Roth B. L. Engineered GPCRs as tools to modulate signal transduction. Physiology (Bethesda) 23, 313–321 (2008). 19074739 10.1152/physiol.00025.2008

[b11] Armbruster B. N., Li X., Pausch M. H., Herlitze S. & Roth B. L. Evolving the lock to fit the key to create a family of G protein-coupled receptors potently activated by an inert ligand. Proc. Natl Acad. Sci. USA 104, 5163–5168 (2007). 17360345 10.1073/pnas.0700293104 PMC1829280

[b12] Ferguson S. M. *et al.* Transient neuronal inhibition reveals opposing roles of indirect and direct pathways in sensitization. Nat. Neurosci. 14, 22–24 (2011). 21131952 10.1038/nn.2703 PMC3058296

[b13] Chang P., Hashemi K. S. & Walker M. C. A novel telemetry system for recording EEG in small animals. J. Neurosci. Methods 201, 106–115 (2011). 21820010 10.1016/j.jneumeth.2011.07.018

[b14] Wulff P. & Arenkiel B. R. Chemical genetics: receptor-ligand pairs for rapid manipulation of neuronal activity. Curr. Opin. Neurobiol. 22, 54–60 (2012). 22119143 10.1016/j.conb.2011.10.008 PMC3294416

[b15] Louis E. D., Williamson P. D. & Darcey T. M. Chronic focal epilepsy induced by microinjection of tetanus toxin into the cat motor cortex. Electroencephalogr. Clin. Neurophysiol. 75, 548–557 (1990). 1693899 10.1016/0013-4694(90)90141-6

[b16] Nilsen K. E., Walker M. C. & Cock H. R. Characterization of the tetanus toxin model of refractory focal neocortical epilepsy in the rat. Epilepsia 46, 179–187 (2005). 15679498 10.1111/j.0013-9580.2005.26004.x

[b17] Cockerell O. C., Rothwell J., Thompson P. D., Marsden C. D. & Shorvon S. D. Clinical and physiological features of epilepsia partialis continua. Cases ascertained in the UK. Brain J. Neurol. 119, (Pt 2): 393–407 (1996). 10.1093/brain/119.2.393 8800935

[b18] Garner A. R. *et al.* Generation of a synthetic memory trace. Science 335, 1513–1516 (2012). 22442487 10.1126/science.1214985 PMC3956300

[b19] Alexander G. M. *et al.* Remote control of neuronal activity in transgenic mice expressing evolved G protein-coupled receptors. Neuron 63, 27–39 (2009). 19607790 10.1016/j.neuron.2009.06.014 PMC2751885

[b20] Baldessarini R. J. *et al.* Tissue concentrations of clozapine and its metabolites in the rat. Neuropsychopharmacology. 9, 117–124 (1993). 8216694 10.1038/npp.1993.50

[b21] Levey A. I., Edmunds S. M., Koliatsos V., Wiley R. G. & Heilman C. J. Expression of m1-m4 muscarinic acetylcholine receptor proteins in rat hippocampus and regulation by cholinergic innervation. J. Neurosci. 15, 4077–4092 (1995). 7751967 10.1523/JNEUROSCI.15-05-04077.1995 PMC6578239

[b22] Shirey J. K. *et al.* An allosteric potentiator of M4 mAChR modulates hippocampal synaptic transmission. Nat. Chem. Biol. 4, 42–50 (2008). 18059262 10.1038/nchembio.2007.55

[b23] Guitton C., Abbar M., Kinowski J. M., Chabrand P. & Bressolle F. Multiple-dose pharmacokinetics of clozapine in patients with chronic schizophrenia. J. Clin. Psychopharmacol. 18, 470–476 (1998). 9864080 10.1097/00004714-199812000-00010

[b24] Hovorka R. Closed-loop insulin delivery: from bench to clinical practice. Nat. Rev. Endocrinol. 7, 385–395 (2011). 21343892 10.1038/nrendo.2011.32

[b25] Cook M. J. *et al.* Prediction of seizure likelihood with a long-term, implanted seizure advisory system in patients with drug-resistant epilepsy: a first-in-man study. Lancet Neurol. 12, 563–571 (2013). 23642342 10.1016/S1474-4422(13)70075-9

[b26] Akam T., Oren I., Mantoan L., Ferenczi E. & Kullmann D. M. Oscillatory dynamics in the hippocampus support dentate gyrus–CA3 coupling. Nat. Neurosci. 15, 763–768 (2012). 22466505 10.1038/nn.3081 PMC3378654

